# Branched-chain amino acid catabolism initiates volatile synthesis in *Gentiana triflora*

**DOI:** 10.1007/s00425-025-04772-4

**Published:** 2025-07-23

**Authors:** Takuya Teshima, Keiichirou Nemoto, Motoki Shimizu, Chiharu Yoshida, Akiko Hirabuchi, Fumina Goto, Takashi Nakasato, Zenbi Naito, Masahiro Nishihara

**Affiliations:** 1 Iwate Biotechnology Research Center, 22-174-4 Narita, Kitakami, Iwate 024-0003 Japan; 2 Iwate Agricultural Research Center, 22-1 Narita, Kitakami, Iwate 024-0003 Japan; 3 Present Address: Department of Bioscience and Biotechnology, Fukui Prefectural University, 1737 4-1-1 Kenjojima, Matsuoka, Eiheiji-Town, Fukui, 910-1195 Japan

**Keywords:** Branched-chain amino acid transferase, Floral scent, Gentian, 3-Methylbutanoic acid, Volatile organic compounds

## Abstract

**Main conclusion:**

This study identified GeBCAT2 as a key gene in catalyzing the first step of branched chain amino acid biosynthesis in Gentiana triflora, thereby contributing to unpleasant floral odor emission.

**Abstract:**

Gentians, widely cultivated as ornamental flowers in Japan, primarily originate from the endemic gentian species *Gentiana triflora* and *G. scabra.* This study analyzed volatile compounds in Japanese gentians using gas chromatography–mass spectrometry. Results showed that *G. triflora* flowers consistently emitted 3-methylbutanoic acid, 2-methylbutanoic acid, and isobutyric acid, which are volatile organic compounds derived from branched-chain amino acids (BCAAs) and associated with unpleasant odors. In contrast, *G. scabra* flowers did not emit these compounds. Although the BCAA metabolism has been widely studied, its catabolic pathways in gentians remain unclear. Therefore, we performed precursor feeding experiments to quantitatively verify the role of BCAAs and their corresponding keto acids in producing odorous volatiles. We also cloned and functionally analyzed two *Gentiana* BCAAs transferase genes (*GeBCAT1* and *GeBCAT2*). Both genes were more highly expressed in flowers than in leaves, with expression levels higher in *G. triflora* than in *G. scabra*. Enzymatic assays with recombinant proteins demonstrated that GeBCAT1 and GeBCAT2 participate in BCAA-related catabolic reactions. Notably, GeBCAT2’s substrate specificity for BCAAs correlated with unpleasant odor intensity in *G. triflora*, suggesting that it serves as the primary enzyme initiating unpleasant odor biosynthesis in gentians. These findings provide valuable insights into volatile biosynthesis in gentians and offer a foundation for breeding cultivars with reduced unpleasant odors.

**Supplementary Information:**

The online version contains supplementary material available at 10.1007/s00425-025-04772-4.

## Introduction

Floral scent is a complex trait determined by a mixture of volatile organic compounds (VOCs) (Knudsen et al. 1993). Plant-produced VOCs mediate interactions with other organisms, playing key roles in plant survival and reproduction (Vainstein et al. 2001). In particular, floral scent serves as critical signal for plant–pollinator communication (Pellmyr and Thien 1986). Similar to floral color and form, scent also has aesthetic value and is an important breeding target in ornamental flowers. However, controlling floral scent in agriculture and horticulture remains challenging owing to the multifaceted interactions among multiple aroma compounds and the complexity of their biosynthetic pathways.

*Gentiana*, a genus in the family Gentianaceae, includes approximately 400 species (Köhlein 1991) primarily distributed in the temperate mountain regions of Asia, Europe, and North and South America. Some *Gentiana* species are cultivated as ornamental plants, including potted plants and cut flowers. Among these, Japanese cultivated gentians, bred from *G. triflora* and *G. scabra*, display vivid blue flowers due to high levels of anthocyanins, such as gentiodelphin (Goto et al. 1982), and are economically important in Japan (Köhlein 1991). However, *Gentiana* flowers emit unpleasant-smelling compounds, making them unsuitable for indoor use (Lee et al. 2010). Their unpleasant odor is primarily attributed to 2-methylbutanoic acid and 3-methylbutanoic acid, which are emitted specifically by *G. triflora*. These VOCs are associated with off-flavors in fermented products, including soy sauce, where they are produced by yeast (Watanabe et al. 2013). In yeast, 2-methylbutanoic acid and 3-methylbutanoic acid are synthesized from isoleucine (Ile) and leucine (Leu) via the branched-chain amino acids (BCAAs) degradation pathway, also known as the Ehrlich pathway (Hazelwood et al. 2008). However, this pathway has not been reported in plants. Although some plants, such as *Gypsophila paniculata* L., also release these VOCs, details of their biosynthesis and ecological significance remain unknown (Nimitkeatkai et al. 2006).

**Fig. 1:**
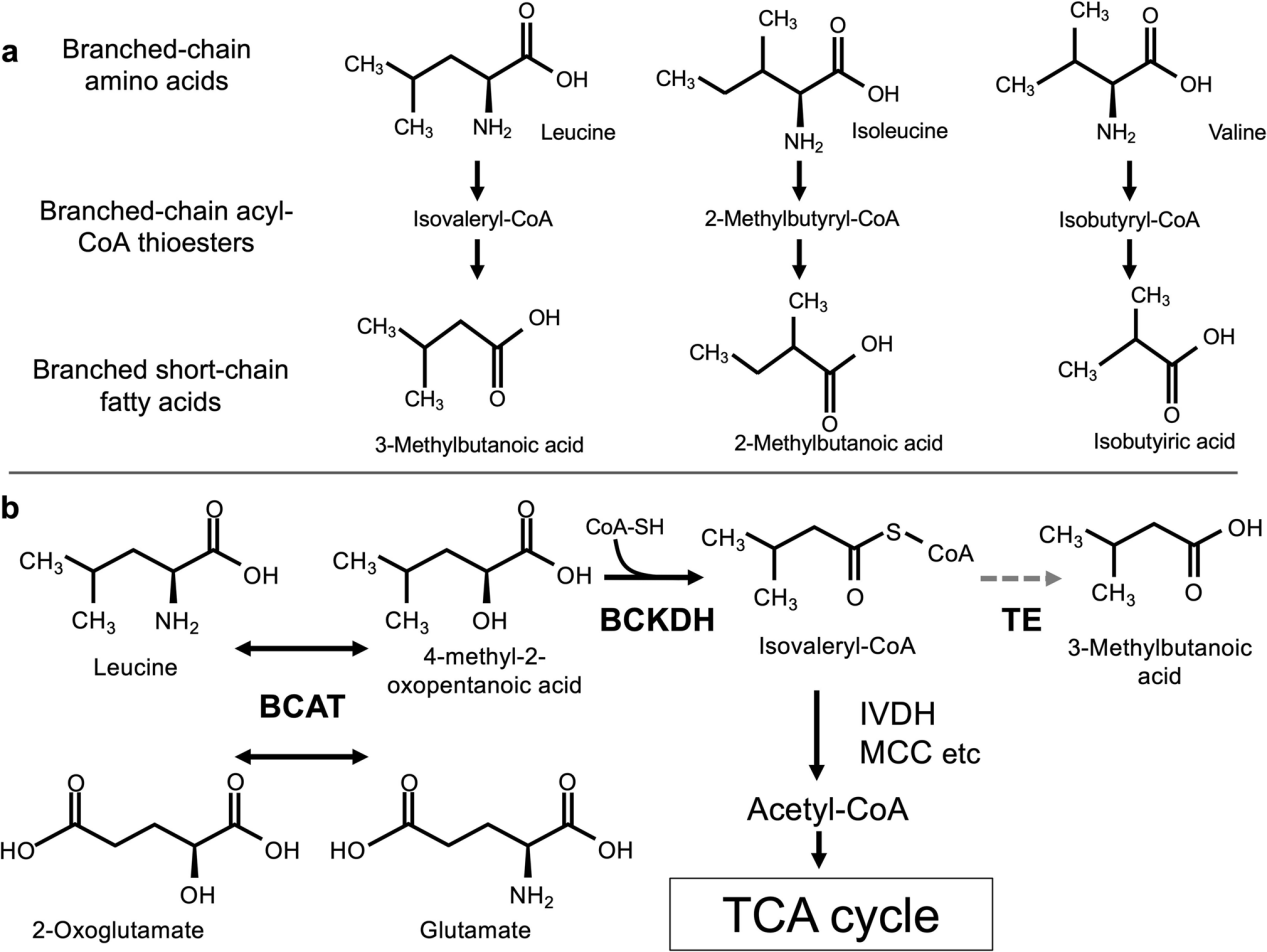
Biosynthesis of VOCs from branched-chain amino acids (BCAAs). **a** 3-Methylbutanoic acid from leucine, 2-methylbutanoic acid from isoleucine, and isobutyric acid from valine via their corresponding acyl-CoA thioesters. **b** Schematic depiction of the major pathways contributing to the biosynthesis of 3-methylbutanoic acid in plants. *BCAT* branched-chain amino acid aminotransferase, *BCKDH* branched-chain keto acid dehydrogenase, *IVDH* isovaleryl-CoA dehydrogenase, *MCC* methylcrotonyl-CoA carboxylase, *TE* thioesterase. Solid arrows represent steps supported by previous study; dashed arrows represent proposed steps yet to be elucidated

In plants, branched-chain acyl-CoA thioesters are essential intermediates in the biosynthesis pathways of secondary metabolites derived from BCAAs such as 3-methylbutanoic acid (also known as isovaleric acid). Isovaleryl-CoA, isobutyryl-CoA, and 2-methylbutyryl-CoA are derived from the degradation of Leu, valine (Val), and Ile, respectively (Fig. 1a). This BCAA degradation occurs in mitochondria (Goese et al. 1999; Araujo et al. 2010), where branched acyl-CoA primarily functions as an intermediate in the tricarboxylic acid cycle. However, a unique pathway is believed to exist in which some acyl-CoAs are converted into branched short-chain fatty acids by a thioesterase (Xu et al. 2013) (Fig. 1b). Despite these insights, many aspects of BCAA catabolism in plants remain unclear.

This study focuses on branched-chain amino acid transferases (BCATs), which play key roles in forming branched-chain acyl-CoAs. BCATs catalyze the final step of BCAA biosynthesis and the initial step of their catabolism (Hagelstein et al. 1997; Homeyer et al. 1985; Binder et al. 2007). Specifically, BCATs link BCAA biosynthesis and degradation, converting Leu to 4-methyl-2-oxopentanoic acid (KIC), Ile to 3-methyl-2-oxopentanoic acid (KMV), and Val to 3-methyl-2-oxobutanoic acid (KIV). In *Arabidopsis thaliana*, six BCATs have been identified, along with six in tomato (*Solanum lycopersicum*), five in rice (*Oryza sativa*), and four in hops (*Humulus lupulus*) (Binder et al. 2007; Maloney et al. 2010; Jin et al. 2019; Clark et al. 2013). In *A. thaliana*, *AtBCAT1* catabolizes all BCAAs across various tissue types (Schuster and Binder 2005), whereas *AtBCAT2* expression is flower-specific and upregulated under stress, and *AtBCAT6* is expressed in flowers and siliques (Peng et al. 2015; Lachler et al. 2015). *AtBCAT3*, *AtBCAT4*, and *AtBCAT5* expression shows less tissue specificity (Liepman and Olsen 2010). In other plant species, some *BCAT*s are expressed in specific organs, such as tomato fruit and hop lupulin, whereas others are expressed in multiple tissues. These findings indicate that *BCAT* gene expression is tissue- and organ-specific in plants.

In this study, we cloned and analyzed BCATs in gentian flowers, identifying two BCAT-encoding genes in *Gentiana*: *GeBCAT1* and *GeBCAT2*. In vitro enzymatic activity assays demonstrated that GeBCAT1 and GeBCAT2 specifically catalyze catabolic reactions. To our knowledge, this is the first report on *BCAT* gene cloning related to VOC biosynthesis in gentians. The study’s findings provide evidence of flower-specific BCAA metabolism producing precursors of unpleasant odors in *G. triflora*. Moreover, this research enhances our understanding of molecular-level BCAA-derived secondary metabolite biosynthesis in plants and offers valuable genetic information for breeding gentians with improved floral scent.

## Materials and methods

### Plant material

Two gentian cultivars, *G. triflora* “Maciry” and *G. scabra* “Alta” were used in this study. For BCAA and BCKA feeding experiments, plants were grown in half-strength Murashige and Skoog (MS) medium supplemented with 3% (w/v) sucrose and 0.2% (w/v) gellan gum, under a 16:8-h light:dark cycle at 20 °C with a light intensity of ~ 30 μmol m^−2^ s^−1^. For next-generation sequencing and quantitative real-time polymerase chain reaction (qRT-PCR) analyses, petals and leaves from “Maciry” and “Alta” were collected from plants cultivated in an experimental field at the Iwate Agricultural Research Center, Kitakami City (E 141°10′, N 39°35′) during 2021 and 2024.

### Determination of VOCs in gentians

*G. triflora* and *G. scabra* were grown in the field, and petals were collected and sealed in 10 mL glass vials. VOCs were extracted using methyl tert-butyl ether (MTBE) for GC–MS analysis. In total, 4 mL of MTBE containing *n*-nonanyl acetate (internal standard, 1 μg mL^−1^) was added, and the mixture was centrifuged at 2870 g for 10 min to collect the upper organic phase. GC–MS was performed using Agilent 6890 N and Agilent 5975B MSD (Agilent Technologies, Inc., Santa Clara, CA, USA) with a 0.25 mm × 30 m DB-WAX column (film thickness 0.50 μm; GL Science Inc., Tokyo, Japan). Samples were injected in splitless mode at 200 °C. The column temperature was initially held at 40 °C for 5 min, increased at 5 °C min^−1^ to 250 °C, and maintained at 250 °C for 2 min. Helium was used as the carrier gas at a flow rate of 48.1 kPa. The injector and interface were maintained at 200 °C, and the mass detector operated in electron impact mode with an ionization energy of 70 eV. 3-Methylbutanoic acid, 2-methylbutanoic acid, isobutyric acid, benzyl alcohol, phenylethyl alcohol, and methyl nicotinate were identified by comparing MS profiles and retention times with authentic standards (Supplementary Fig. S1). When required, fragment ions (*m/z* 60 and *m/z* 57) were monitored for high-specificity detection of 3-methylbutanoic acid and 2-methylbutanoic acid. 3-Methylbutanoic acid, 2-methylbutanoic acid, and isobutyric acid were quantified using externally constructed calibration curves. Lilac aldehyde and lilac alcohol were identified using the NIST08s database.

### Metabolite feeding

Petals at developmental stage 4 (as shown in Supplementary Fig. S2) were collected, immediately soaked in 5 mL of 3 mM BCAAs, BCKAs or water, and incubated at 25 °C for 12 h under light exposure. To determine VOCs, petals were carefully washed, calyces were gently removed, and samples were weighed and placed in 10 mL glass tubes. Subsequently, VOCs were extracted using methyl tert-butyl ether (MTBE) and analyzed as described earlier.

### RNA sequencing analysis

Total RNA was isolated from petals (developmental stage S1–S4) and leaves of “Maciry” for de novo transcriptome assembly. Library construction, sequencing, and assembly were performed as described by Takase et al. (2022). RNA-seq data were deposited in the DNA Data Bank of Japan. The data have been deposited with links to BioProject accession number PRJDB20454 in the DDBJ BioProject database.

### qRT-PCR analysis of gentian petals and leaves

Total RNA was extracted from petals (S1-S4) and leaves using the Plant Total RNA Pruifcation Kit (COSMO Bio Co., Ltd., Tokyo, Japan). First-strand cDNA was synthesized from 500 ng of total RNA using ReverTra Ace^®^ qPCR RT Master Mix with gDNA Remover (Toyobo, Osaka, Japan). Relative expression levels of endogenous *GeBCAT1* and *GeBCAT2* were analyzed via qRT-PCR (QuantStudio™ 3 Real-Time PCR, Thermo Fisher Scientific). Expression values were calculated using the 2^*−ΔΔ*Ct^ method and normalized to the gentian ubiquitin 2 (*GeUBQ2*) gene as an internal control. Primer sets are listed in Table S1.

### Subcellular localization analysis of GeBCATs

To investigate the subcellular localization of GeBCAT1 and GeBCAT2, petal protoplasts were prepared as described by Nemoto et al. (2022). Briefly, petals were sandwiched between polyvinyl chloride tape, and the adaxial and abaxial epidermal cell layers were peeled off and collected by carefully separating the tape. The isolated protoplasts were resuspended in MaMg buffer [5 mM MES (pH 5.7), 0.4 M mannitol, and 15 mM MgCl_2_]. In total, 15 μg of p35SΩ-GW-V5-GeBCAT1-YFP-NOST  or p35SΩ-GW-V5-GeBCAT2-YFP-NOST plasmid DNA was transfected into 2 × 10^5^ protoplasts using the PEG-mediated method (Wu et al. 2009), followed by resuspension in WI buffer [4 mM MES (pH 5.7), 0.5 M mannitol, and 20 mM KCl]. After overnight incubation in the dark at 22 °C, cells were observed under a fluorescent microscope (Leica) with a 40 × objective. Mitochondria were stained with 20 nM MitoRed (DOJINDO, Kumamoto, Japan) to analyze GeBCAT1 localization, whereas chloroplast autofluorescence was used to assess GeBCAT2 localization.

### Production of recombinant proteins by wheat cell-free protein synthesis

Total RNA was extracted from petals and leaves (five flower and leaf samples) using the Plant Total RNA Pruifcation Kit (COSMO Bio). First-strand cDNA was synthesized from 500 ng of total RNA using ReverTra Ace^®^ qPCR RT Master Mix with gDNA Remover (Toyobo). The open-reading frames of *GeBCAT1* and *GeBCAT2* in *G. triflora* “Maciry” were amplified using PrimeSTAR Max DNA Polymerase (Takara), and the amplification products were cloned into pDONR221 (Invitrogen) and recombined into pEU-FLAG-GST-GW (Yamanaka et al. 2023) using LR Clonase (Invitrogen). In vitro transcription and translation were performed using the bilayer method with the WEPRO7240 Expression Kit (Cell-Free Sciences, Ehime, Japan) according to the manufacturer’s instructions. Synthesized recombinant proteins were purified using MagneGST™ Protein Purification System (Promega) following the manufacturer’s protocol. Purified proteins were used for expression analysis and functional characterization. Primer sets are listed in Table S1.

### Enzyme assays using recombinant proteins

Enzyme assays for GeBCAT1 and GeBCAT2 were performed as described previously (Schuster and Binder 2005; Schuster et al. 2006). For deamination assays, reactions were conducted in a total volume of 50 μL containing 0.1 M Tris–HCl (pH 8.0), 120 mM α-ketoglutaric acid, 0.16 mM pyridoxale-5-phosphate (PLP), 4 mM DTT, 8 μg of recombinant proteins, and varying substrate concentrations (0–10 mM Val, 0–5 mM Leu, or 0–5 mM Ile). For amination assays, the enzyme reactions were performed in a total volume of 50 μL containing 0.1 M Tris–HCl (pH 8.0), 150 mM L-glutamate, 0.16 mM PLP, 4 mM DTT, 8 μg of recombinant proteins, and various substrate concentrations (0–5 mM KIC, 0–5 mM KMV, or 0–5 mM KIV.) Reaction mixtures were incubated at 33 °C for 20 min, terminated at 94 °C for 10 min before liquid chromatography–time-of-flight mass spectrometry (LC–TOF–MS) analysis (Noguchi et al. 2014). Reaction velocity was correlated with substrate concentration, following typical Michaelis–Menten kinetics, with data fitted to a nonlinear regression curve using Hyper32 (https://hyper32.software.informer.com).

### Derivatization procedure of samples and LC–TOF–MS conditions

An *O*-(2,3,4,5,6-pentafluorobenzyl) hydroxylamine (PFBHA) hydrochloride solution (10 mg/mL) was prepared by dissolving PFBHA in a 1:1 (v/v) mixture of acetonitrile and 0.1% (wt/wt) NaOH. To derivatize keto acids, 20 μL of each sample was mixed with 20 μL of 10 mg/mL PFBHA solution and incubated at 0 °C (ice water) for 60 min. The derivatization reaction was quenched with 10 μL of a 1:1 (v/v) acetonitrile and acetone mixture. Samples were stored at 4 °C in the auto-sampler of the LC system until LC–TOF–MS analysis.

LC–TOF–MS analysis was performed on a Sciex HPLC Prominence System (Sciex) equipped with an AB Sciex TripleTOF^®^ 5600 + System. The auto-sampler temperature and injection volume were set to 4 °C and 4 µL, respectively. Liquid chromatography was performed at 40 °C using a Unison UK-Phe column (150 × 2 mm, 3 µm; Imtakt Corporation, Kyoto, Japan) and a gradient elution system with a mobile phase comprising solvent A (water/formic acid, 100:0.1, v/v) and solvent B (acetonitrile/formic acid, 100:0.1, v/v). A 400 µL min^−1^ flow rate was maintained throughout the analysis. The gradient elution conditions were as follows: 0–4 min (10% B), 8 min (35% B), 20 min (40% B), 30–35 min (95% B), and 40 min (10% B), followed by a return to the initial conditions (10% B) for 5 min (equilibration) before the next sample injection. The mass spectrometer was operated in enhanced MS scan mode with negative detection. Ionization was performed via electrospray ionization, with the following parameters: ion source temperature: 300 °C; curtain gas: 40 psi; ion source gas: 50 psi; collision gas: 5 psi; ion source voltage: − 4200 V; entrance potential: − 10 V.

## Results

### Analysis of VOCs in gentian petals

**Fig. 2:**
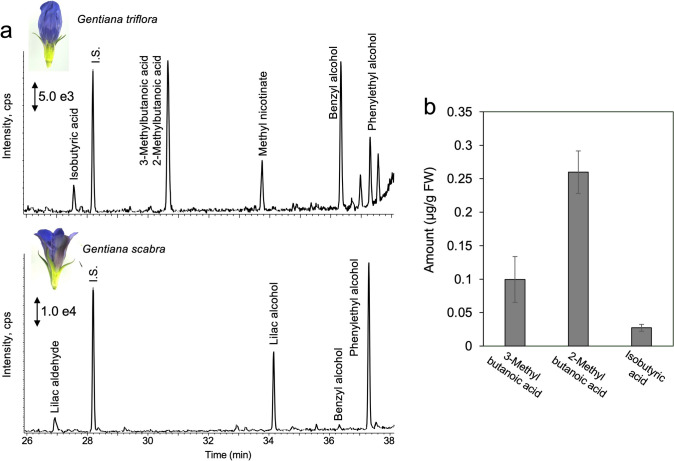
Identification of VOCs emitted from *Gentiana* flowers. **a** Representative chromatograms of volatiles emitted from S4 stage petals of *G. triflora* and *G. scabra*. **b** Quantitative analysis of 3-methylbutanoic acid, 2-methylbutanoic acid, and isobutyric acid, branched-chain VOCs of “Maciry.” Data are presented as means ± SDs (*n* = 3)

To investigate the VOCs in gentian flowers, we analyzed the floral scent profiles of *G. triflora* “Maciry” and *G. scabra* “Alta” using GC–MS (Fig. 2a). In “Maciry,” volatiles with unpleasant odors, including 3-methylbutanoic acid, 2-methylbutanoic acid, and isobutyric acid, were detected (Fig. 2b). In contrast, “Alta” emitted terpenoid compounds, such as lilac aldehydes and lilac alcohols; however, no unpleasant floral scents were identified (Fig. 2a). Among the unpleasant-smelling volatiles emitted by *G. triflora*, 2-methylbutanoic acid was the most abundant, followed by 3-methylbutanoic acid and then isobutyric acid. The production of these compounds peaked at floral development stage 4, when the petals were fully open (Supplementary Fig. S2). Further analysis of different flower organs revealed that these volatiles were most concentrated at the base of the petals, where nectar accumulates (Supplementary Fig. S3). The presence of 3-methylbutanoic acid was also assessed across 41 gentian cultivars and breeding lines, including “Maciry” and “Alta.” Although *G. triflora* cultivars varied in their 3-methylbutanoic acid levels, none of the *G. scabra* cultivars contained detectable amounts, demonstrating a clear species-specific difference (Supplementary Fig. S4).

### Substrate feeding in G. triflora petals

**Fig. 3:**
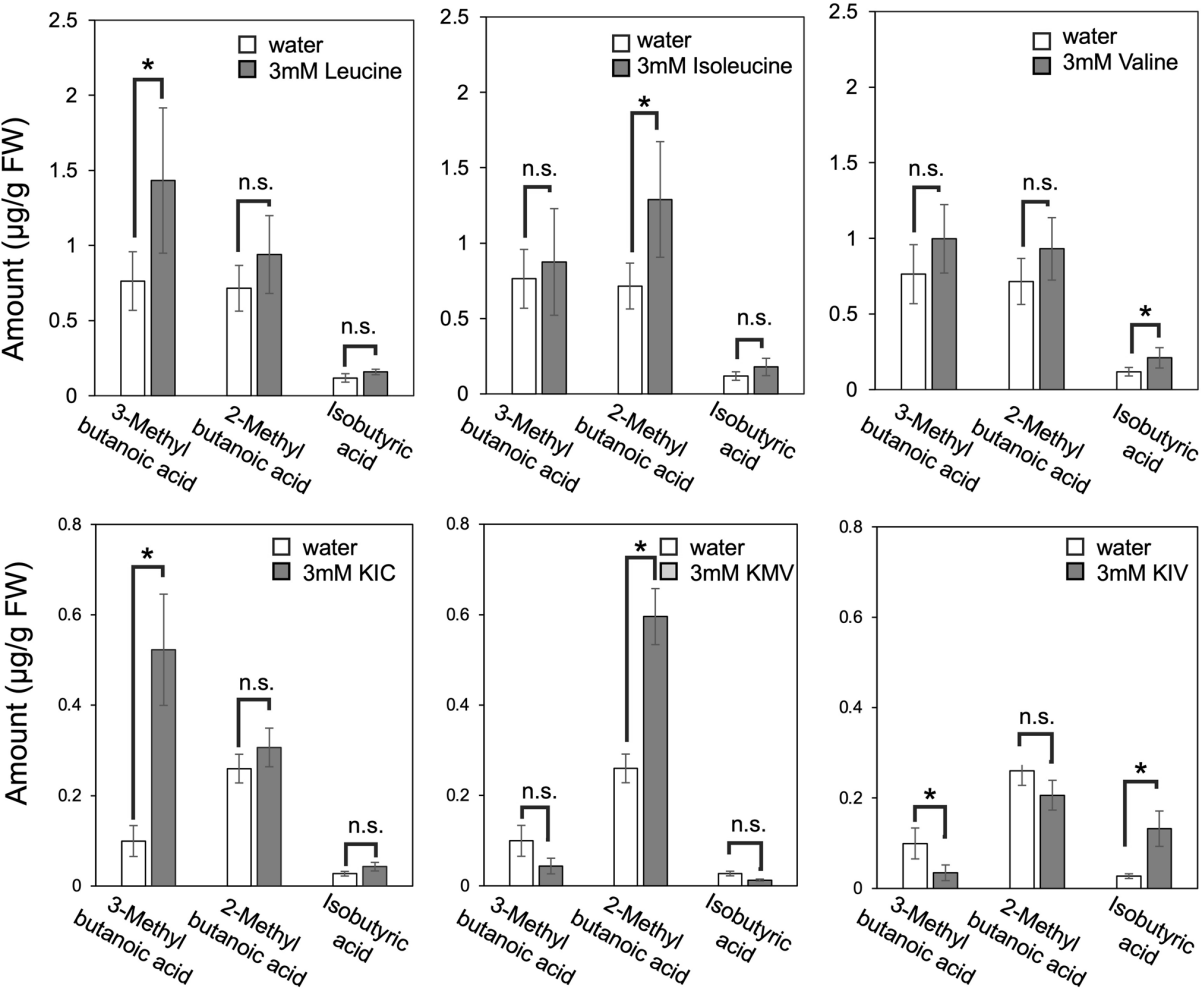
Volatile production following branched-chain substrate feeding in *G. triflora* petals. Changes in branched-chain VOCs after feeding petals with the specified BCAA (leucine, isoleucine, or valine) or BCKA (KIC, KMV, or KIV) substrates and overnight incubation. Values are expressed as the percentage of volatile emission relative to water-fed controls ± SDs (*n* = 3). Columns marked with an asterisk indicate statistically significant changes based on Student’s *t* test (*: 0.01 < *P* < 0.05, n.s.: 0.05 < *P*)

To explore the relationship between precursors and volatile biosynthesis, petals of *G. triflora* were fed with each BCAA and BCKA, which are potential precursors of branched-chain volatiles (Fig. 1). Emission levels of volatile compounds were then quantified and compared to water-fed controls. Based on side-chain chemistry, 3-methylbutanoic acid is hypothesized to derive from Leu and KIC, 2-methylbutanoic acid from Ile and KMV, and isobutyric acid from Val and KIV (Fig. 1a). Consistent with this hypothesis, Leu and KIC supplementation led to significant increases in 3-methylbutanoic acid of approximately 2- and fivefold, respectively (Fig. 3). Similarly, Ile and KMV feeding resulted in significant increases in 2-methylbutanoic acid of approximately 1.8- and 2.3-fold, respectively (Fig. 3), and Val and KIV supplementation led to significant increases in 2-methylbutanoic acid of about 1.8- and 4.9-fold, respectively (Fig. 3). Notably, branched-chain VOCs increased only when the corresponding BCAAs and BCKAs were supplied as substrates. An unexpected decrease in 3-methylbutanoic acid was observed upon KIV feeding. This reduction is likely due to the metabolic interconnection between Leu and Val biosynthetic pathways, as previously suggested (Hagelstein et al. 1997; Homeyer et al. 1985). These findings confirm that BCAAs and BCKAs play a crucial role in the biosynthesis of branched-chain volatiles in *G. triflora*.

### Cloning and characterization of GeBCAT genes

To identify genes involved in branched-chain volatile biosynthesis, we performed de novo transcriptome assembly on gentian petals at various developmental stages and leaves of *G. triflora* “Maciry.” This analysis led to the identification of two BCAT homologs: *GeBCAT1*, expressed specifically in flowers, and *GeBCAT2*, expressed across all tissues. Full-length cDNA sequences of both genes were cloned and analyzed. GeBCAT1 and GeBCAT2 encode 390- and 411-amino-acid proteins, respectively, which share 62% sequence identity and 86% homology. Notably, the amino acid sequences of GeBCAT1 and GeBCAT2 are identical in “Maciry” and “Alta” (Supplementary Fig. S5). TargetP-2.0 analysis (http://www.cbs.dtu.dk/services/TargetP/) predicted that GeBCAT1 contains a mitochondrial transit peptide, whereas GeBCAT2 contains a plastid transit peptide.

**Fig. 4:**
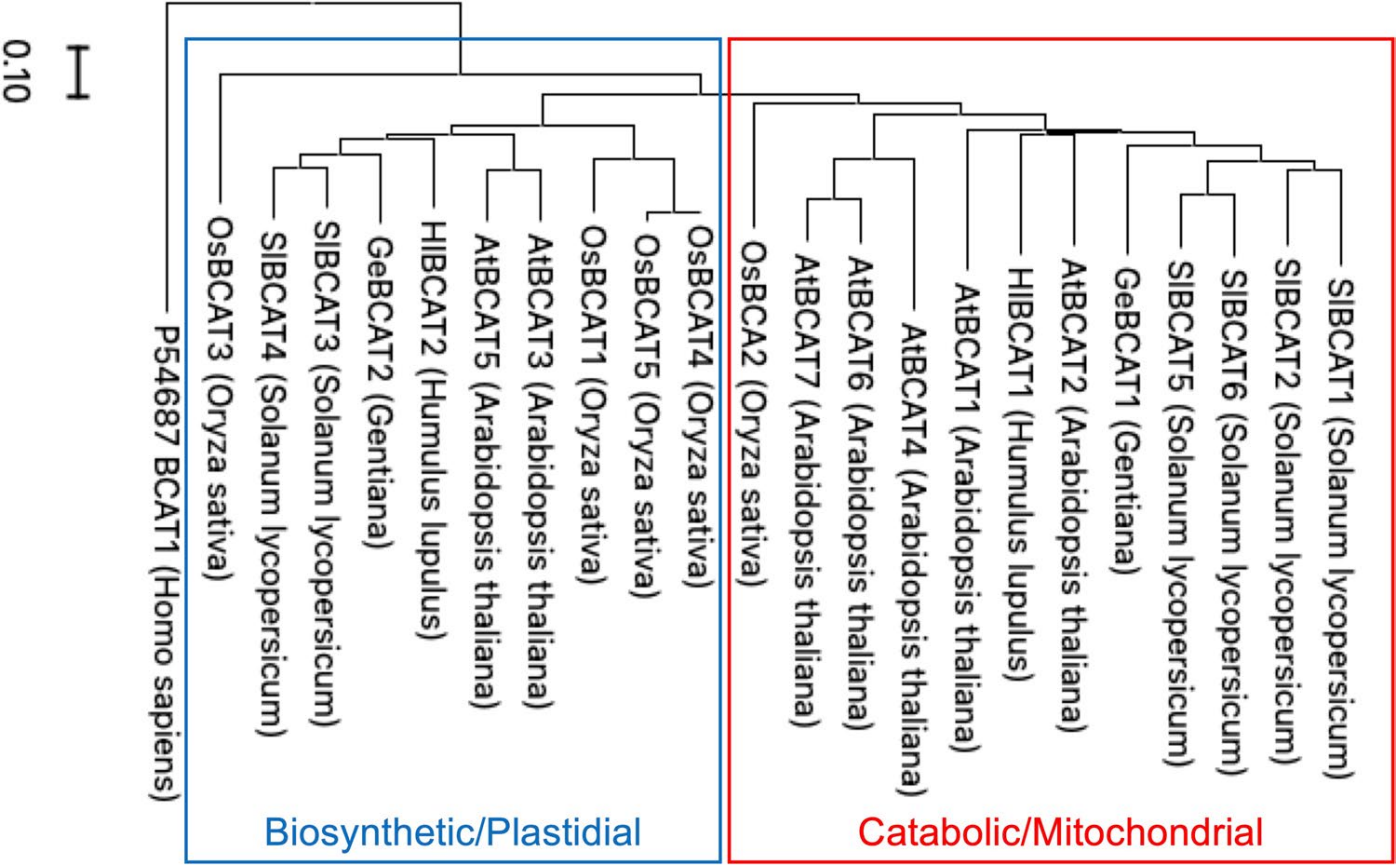
Characterization of GeBCAT1 and GeBCAT2 sequences. Phylogenetic analysis of GeBCAT1 and GeBCAT2 with BCAT-related proteins from different plant species. Phylogenetic tree was constructed using MEGA X with the neighbor-joining method and 1000 bootstrap replicates. Numbers at internal nodes represent support values from the bootstrap analysis. Scale bar: 0.1 changes per amino acid position. Blue box indicates the biosynthetic/plastidial clade; red box indicates the catabolic/mitochondrial clade

A phylogenetic tree was constructed using BCAT protein sequences from hop, *Arabidopsis*, and tomato obtained from the Phytozome database (Goese et al. 1999; Hazelwood et al. 2008; Maloney et al. 2010; Clark et al. 2013). The analysis grouped BCAT proteins into two distinct clades: one associated with biosynthetic/plastidial function and the other with catabolic/mitochondrial function (Fig. 4). GeBCAT1 clustered with the catabolic clade, whereas GeBCAT2 clustered with the biosynthetic clade. Sequence alignments revealed that gentian BCAT proteins contain conserved amino acid residues found in other species’ BCATs (Supplementary Fig. S6), including a pyridoxal–phosphate linking site and functionally essential sites previously identified in *Escherichia coli* and *Homo sapiens* crystal structures (Okada et al. 1997; Yennawar et al. 2001).

The subcellular localization of GeBCAT1 and GeBCAT2 was analyzed via transient expression in petal protoplasts. C-terminal YFP fusion constructs of GeBCAT1 and GeBCAT2 were introduced into *G. triflora* petal protoplasts and observed using fluorescent microscopy. GeBCAT1 localized to the mitochondria, consistent with predictions from TargetP-2.0 and phylogenetic analysis (Supplementary Fig. S7). Notably, gentian flower petals contain functional chloroplasts in specific epidermal regions (Takahashi et al. 2020). GeBCAT2, predicted to localize to plastids, was particularly detected in these chloroplasts (Supplementary Fig. S8).

**Fig. 5:**
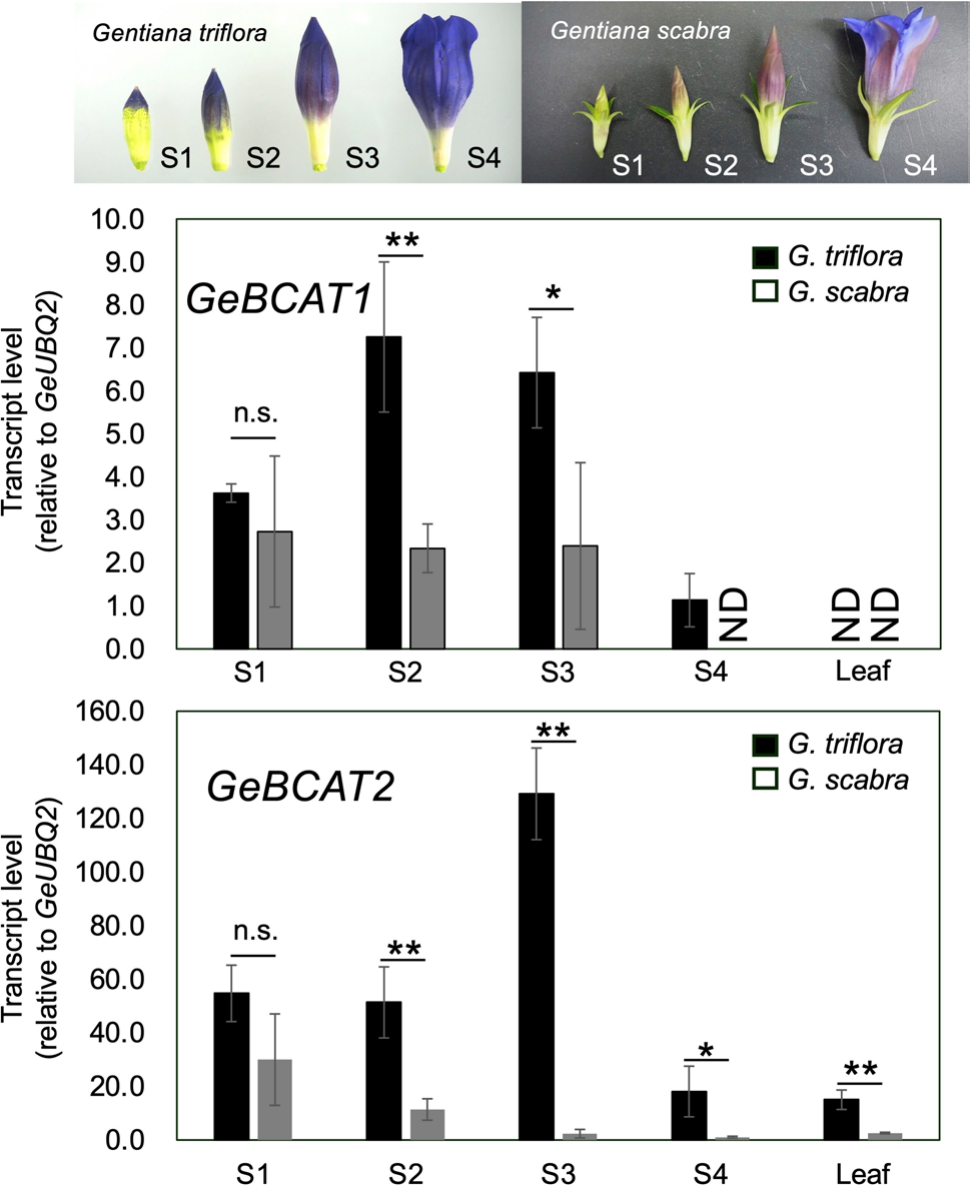
*GeBCAT1* and *GeBCAT2* expression in petals and leaves. Ubiquitin 2 (*GtUBQ2*) was used as the internal control in qRT-PCR analyses, and expression levels relative to this control are shown as multiples of the lowest value of 1. ND, not detectable. Data are presented as means ± SDs (*n* = 3). Asterisks marking columns indicate statistically significant changes based on Student’s t test (**: *P* < 0.01, *: 0.01 < *P* < 0.05)

To examine *GeBCAT1* and *GeBCAT2* expression profiles, qRT-PCR analysis was performed in flowers and leaves. No significant difference in *GeBCAT1* expression was observed at petal development stage 1 between *G. triflora* and *G. scabra*; however, *G. triflora* showed 3.1- and 2.7-fold higher expression at stages 2 and 3, respectively (Fig. 5). *GeBCAT1* transcripts were undetectable in *G. scabra* at stage 4 and in the leaves of both species. Similarly, *GeBCAT2* expression levels did not significantly differ between species at stage 1, but *G. triflora* exhibited significantly higher expression at stages 2, 3, and 4, increasing by 4.5-, 54.8-, and 17.4-fold, respectively (Fig. 5). Additionally, *GeBCAT2* transcript levels in *G. triflora* leaves were 5.8-fold higher than those in *G. scabra* (Fig. 5).

### Enzymatic activities of GeBCAT proteins in BCAA degradation

**Fig. 6:**
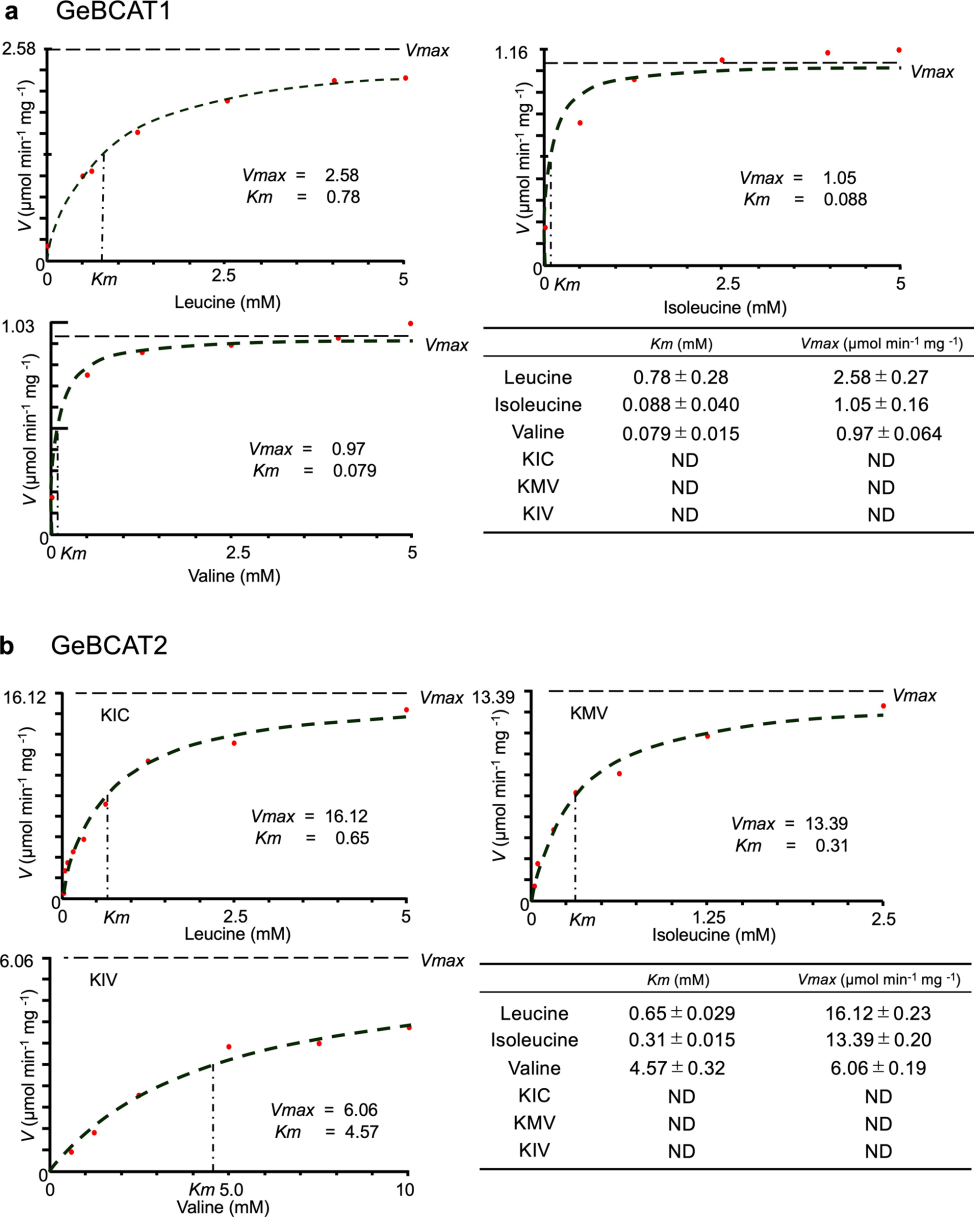
Functional analysis of GeBCATs in vitro. Enzyme activity of GeBCAT1 (**a**) and GeBCAT2 (**b**). Kinetic parameters were assayed with varying concentrations of BCAAs (leucine, isoleucine, or valine). *K*_m_ and *V*_max_ values of recombinant GeBCAT1 and GeBCAT2 are shown for different substrates. KIV 3-methyl-2-oxobutanoate, KMV 3-methyl-2-oxopentanoate, KIC 4-methyl-2-oxopentanoate, *ND* not detectable

To investigate the functional roles of GeBCAT1 and GeBCAT2 in volatile biosynthesis in *G. triflora*, in vitro enzyme assays were performed using recombinant proteins. Both enzymes were assayed for their ability to catalyze transamination reactions in the forward (BCAA synthesis) and reverse (BCAA degradation) directions. The assays showed that GeBCAT1 and GeBCAT2 catalyzed reactions involving Leu, Ile, and Val (Supplementary Fig. S9a) but did not exhibit activity toward BCKAs (Supplementary Fig. S9b). Kinetic analyses revealed that GeBCAT1 and GeBCAT2 followed Michaelis–Menten kinetics. GeBCAT1 displayed a higher affinity for Val and Ile than for Leu (Fig. 6a), whereas GeBCAT2 exhibited a greater affinity for Ile and Leu than for Val (Fig. 6b). Compared with GeBCAT1, GeBCAT2 showed higher specific activity toward BCAAs.

## Discussion

### Differences in volatile components among gentian cultivars

Japanese cultivated gentians are primarily bred from *G. triflora* and *G. scabra*. We analyzed VOCs emitted by flowers from various cultivars and breeding lines in these species. Results revealed that significant differences in volatile compound compositions between the two species. Specifically, *G. scabra*, represented by “Alta,” primarily emitted monoterpenes, such as lilac aldehyde and lilac alcohol (Fig. 2a), whereas *G. triflora* emitted branched-chain volatiles, including 3-methylbutanoic acid, 2-methylbutanoic acid, and isobutyric acid, which are associated with unpleasant odors (Fig. 2a). Other cultivars/lines from *G. scabra* and *G. triflora* displayed similar volatile profiles, although the concentrations varied across lines, as confirmed by 3-methylbutanoic acid levels (Supplementary Fig. S4). Pollinators have been shown to contribute markedly to the diversification of floral scents, with previous studies indicating that scent compounds in flowers influence pollinator behavior in several plant species, including orchids (Peakall et al. 2010; Xu et al. 2011; Whitehead and Peakall 2014). In *Gentiana leucomelaena*, bees are attracted to specific flower traits (Mu et al. 2011). However, the role of floral volatiles and pollinators in Japanese cultivated gentians remains underexplored. Therefore, future studies should investigate the role of these volatiles in pollinator attraction to better understand the ecological context of these compounds.

### Biosynthetic pathway of branched-chain VOCs in gentians

Branched-chain volatile compounds are important flavor volatiles in fruits and vegetables, such as melon and tomato, with both BCAAs and BCKAs serving as key precursors (Gonda et al. 2010; Kochevenko et al. 2012). The substrate feeding experiments in this study demonstrated that BCAAs and BCKAs are key precursors to branched-chain volatiles in gentians, as shown in the previous studies. Our data support a model where KIC and Leu are precursors to 3-methylbutanoic acid, KMV and Ile are precursors to 2-methylbutanoic acid, and KIV and Val are precursors to isobutyl acid (Fig. 3).

Humulone, derived from BCAAs, is the secondary metabolite found in hops, and its intermediate metabolite is known to be isovaleryl-CoA (Xu et al. 2013). The observed increase in 3-methylbutanoic acid following isovaleryl-CoA supplementation to petals suggests that the catabolic pathway of BCAAs contributes to the production of gentian VOCs (Supplementary Fig. S10). Additional candidate genes potentially encoding the aforementioned enzymes are present in the RNA-seq data of “Maciry.” However, their functionality and specific roles in aroma compound biosynthesis in *G. triflora* remain unclear. Transcription factors regulating the biosynthetic pathway may also contribute to the metabolic differences observed between the two species. SNPs or indels in coding or promoter regions of VOC-related genes may influence metabolic variation, not only between species but also among cultivars. Therefore, further studies are needed to fully understand the VOC biosynthetic pathway in gentians.

### Characteristics of BCATs in plants and their potential role in the production of unpleasant odors in gentians

Previous kinetic studies of BCATs in higher plants have shown that these enzymes are typically specialized for the biosynthesis of BCAAs rather than their catabolic degradation. For example, *Arabidopsis* AtBCAT1, a catabolic enzyme, exhibits a preference for the biosynthetic reaction, as indicated by its lower *K*_m_ values for BCKAs (KIC: 0.84 mM) compared with those for BCAA substrates (Lue: 1.66 mM) (Schuster et al. 2006). Additionally, in AtBCAT1, *K*_m_ values for keto acids are lower than those reported in the biosynthetic enzyme AtBCAT3 (Knill et al. 2008). Similarly, *K*_*m*_ values for keto acids are low in the BCAA-degrading enzyme HlBCAT1 from hops, indicating its preference for the forward (biosynthetic) reaction, unlike the synthetic enzyme HlBCAT2 (Clark et al. 2013). In contrast, our study revealed that both GeBCAT1 and GeBCAT2 in gentians are specialized for catabolic reactions. In particular, GeBCAT2 exhibited enzymatic properties similar to those of OsBCAT2 in rice, an enzyme involved exclusively in degradation reactions, with the kinetic traits of GeBCAT2 and OsBCAT2 also being comparable (Sun et al. 2024). Furthermore, the *K*_m_ values for GeBCAT2 follow the order Ile > Leu > Val, aligning with the composition of branched-chain VOCs found in *G. triflora* (Fig. 2b). GeBCAT2 is more highly expressed in *G. triflora* than in *G. scabra*, with its expression being more pronounced in petals than in leaves. The preference of GeBCAT2 for BCAA degradation may contribute to the differences in VOC composition between *G. triflora* and *G. scabra*. Therefore, GeBCAT2, rather than GeBCAT1, is thought to be responsible for the production of unpleasant odors in *G. triflora*. Intracellular localization analysis in gentian petal protoplasts revealed that GeBCAT2 is localized in chloroplasts, which are particularly developed in petal epidermal cells. Takahashi et al. (2020) previously reported the presence of functional chloroplasts in restricted regions of gentian petals, although their biological role remained unclear. Our findings suggest that these chloroplasts may be functionally associated with VOC production in gentian petals. Conversely, GeBCAT1, which is localized in mitochondria, is likely involved in energy metabolism, as indicated through TargetP analysis and subcellular localization analysis. Similarly, AtBCAT2, which is localized in mitochondria, is upregulated under stress conditions in flowers; however, 3-methylbutanoic acid has not been detected among the aromatic components of *Arabidopsis* flowers. Additionally, BCATs that are highly expressed in flowers are known to have hormone regulatory functions, suggesting that these enzymes may plays roles beyond amino acid metabolism (Gao et al. 2009). The gentian BCATs identified in this study are specialized for the catabolic breakdown of BCAAs, in contrast to previously analyzed BCATs in higher plants, which are also involved in biosynthesis. Further investigation is required to achieve a comprehensive understanding of BCAT functions in gentians.

## Conclusion

This study analyzed VOCs emitted by the flowers of Japanese cultivated gentians, bred from *G. triflora* and *G. scabra*, and identified species-specific volatile profiles. Compounds, including 3-methylbutanoic acid, 2-methylbutanoic acid, and isobutyric acid, which are derived from BCAAs, were detected exclusively in *G. triflora* and found to contribute to its characteristic unpleasant odor. The two BCATs identified in this study appear specialized for BCAA degradation, with notably higher expression levels in *G. triflora* petals compared to *G. scabra*. In vitro enzymatic activity assays, along with gene expression profiles, suggest that GeBCAT2, rather than GeBCAT1, is primarily responsible for the unpleasant odor produced by *G. triflora*. These findings provide valuable insights into the enzymes and regulatory proteins involved in the production of unpleasant odors in gentians. A better understanding of this biosynthetic pathway will contribute to future breeding programs aimed at developing gentian cultivars with reduced undesirable odors.

## Supplementary Information

Below is the link to the electronic supplementary material.

Supplementary file1 (PDF 2634 KB)

## Data Availability

All data supporting the findings of this study are available within the paper and its Supplementary Information. The RNA-seq data in this study have been deposited in the DNA Databank of Japan (BioProject accession no. PRJDB20454).

## Abbreviations

BCAAs: Branched-chain amino acids
BCKAs: Branched-chain α-keto acids
BCAT: Branched-chain amino acid transferase
Ile: Isoleucine
KIC: 4-Methyl-2-oxopentanoic acid
KIV: 3-Methyl-2-oxobutanoic acid
KMV: 3-Methyl-2-oxopentanoic acid
Leu: Leucine
Val: Valine
VOCs: Volatile organic compounds
