# Supplementary material for: Branched-chain amino acid catabolism initiates volatile synthesis in Gentiana triflora
Source: Planta. 2025 Jul 23;262(3):62. doi: 10.1007/s00425-025-04772-4 (PMC12287140; doi:10.1007/s00425-025-04772-4)
Supplement: Supplementary file 1 — Supplementary file1 (PDF 2634 KB) [file 425_2025_4772_MOESM1_ESM.pdf]

Supplementary Information

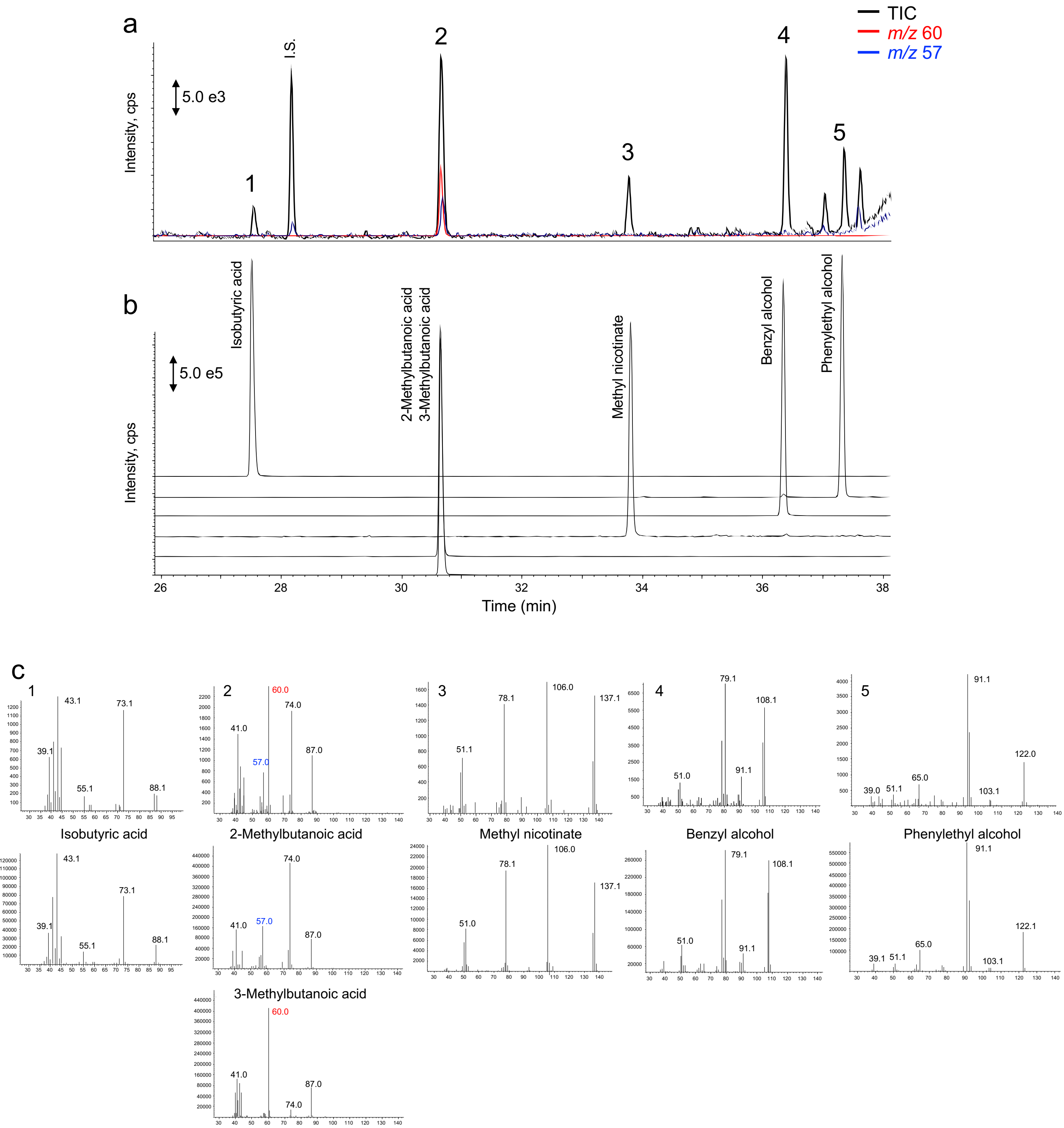

Supplementary Fig. S1 Extended GC-MS analysis of VOCs emitted from *G. triflora.* **a** Representative GC-MS chromatogram of volatile compounds emitted from stage 4 (S4) petals of *G. triflora*. **b** GC-MS chromatograms of authentic standards: isobutyric acid, 2-methylbutanoic acid, 3-methylbutanoic acid, methyl nicotinate, benzyl alcohol and phenylethyl alcohol. **c** Mass spectra of volatile compounds emitted by *G. triflora,* compared with those of authentic standards. The retention times of 2-methylbutanoic acid and 3-methylbutanoic acid were identical due to their structural isomerism; however, they were distinguished based on their characteristic fragment ions at *m/z* 57 and m/z 60, respectively. The black line represents the total ion chromatogram (TIC), the red line *m/z* 60, and the blue line *m/z* 57

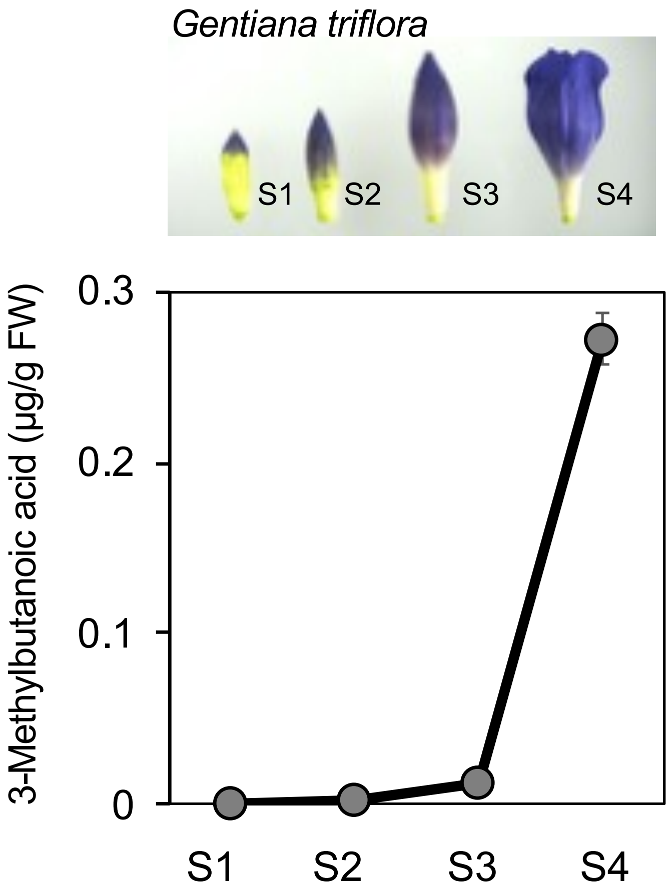

Supplementary Fig. S2 Changes in 3-methylbutanoic acid formation with growth in *G. triflora*.

3-Methylbutanoic acid levels in the petals (S1-S4) of "Maciry" are shown. VOCs were extracted using MTBE for GC-MS analysis. Data are presented as means ± SDs (n = 3)

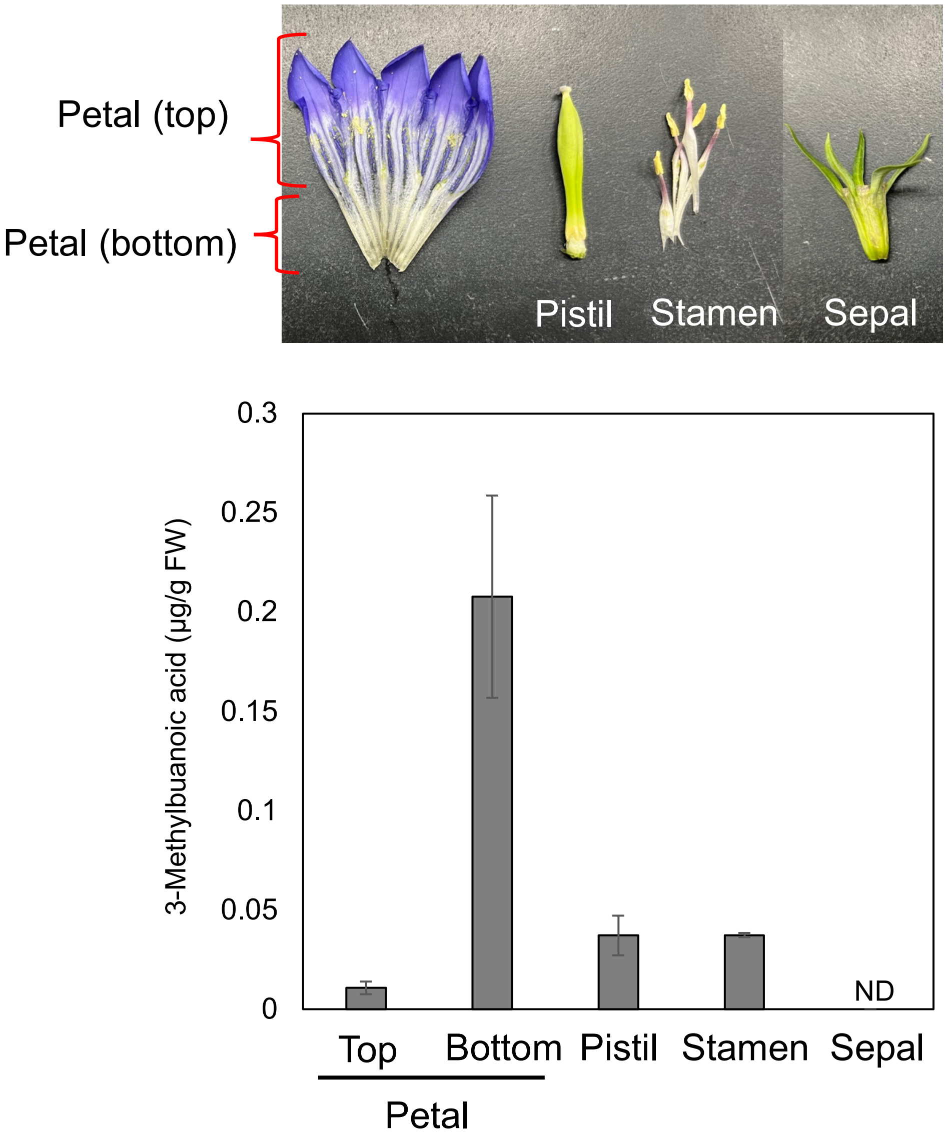

Supplementary Fig. S3 Analysis of 3-methylbutanoic acid released from *G. triflora* flower organs.

3-Methylbutanoic acid levels in the petals (top and bottom regions), pistil, stamens, and sepals of “Maciry” are shown. VOCs were extracted using MTBE for GC-MS analysis. ND, not detectable. Data are presented as means ± SDs (n = 3)

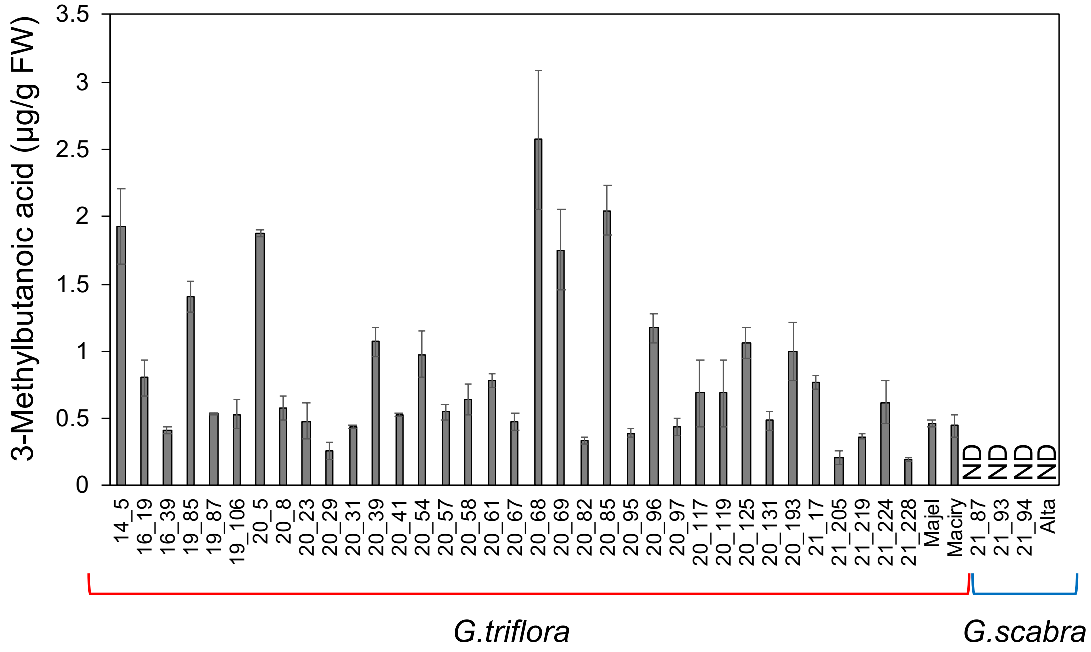

Supplementary Fig. S4. Analysis of aromatic components in gentian cultivars/lines grown in fields at the Iwate Prefectural Agricultural Experiment Station.

3-Methylbutanoic acid levels in 37 *G. triflora* and 4 *G. scabra* lines are shown. VOCs were extracted using MTBE for GC-MS analysis. ND, not detectable. Data are presented as means ± SDs (n = 3)

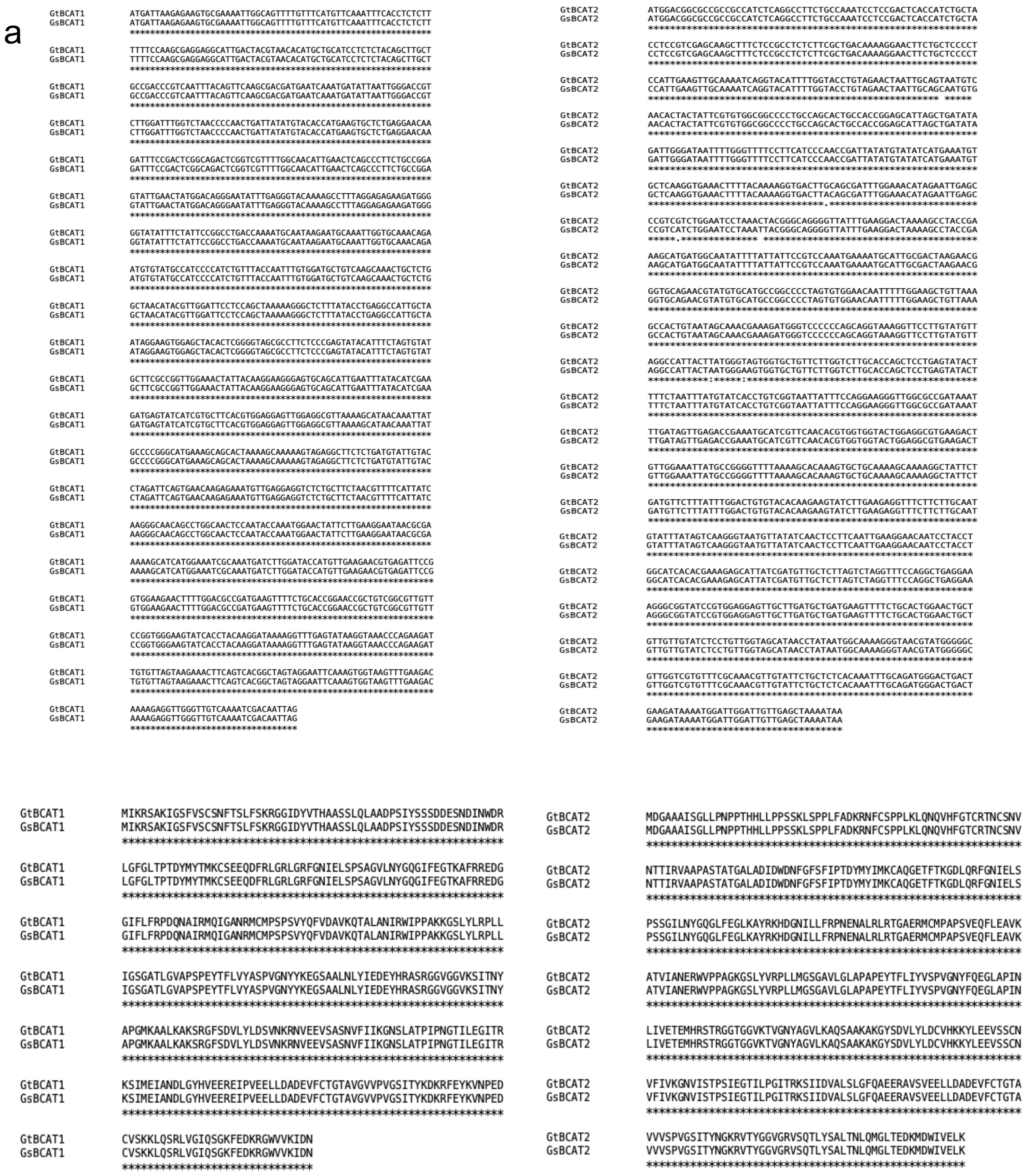

Supplementary Fig. S5

BCAT nucleotide and amino acid sequences from *G. triflora* and *G. scabra*.

a Nucleotide sequences of BCAT genes. **b** Deduced amino acid sequences of BCATs. GtBCAT represents *G. triflora* “Maciry”, and GsBCAT represents *G. scabra* “Alta”. The nucleotide sequences were obtained using a DNA sequencer from cDNA cloned from petal tissues

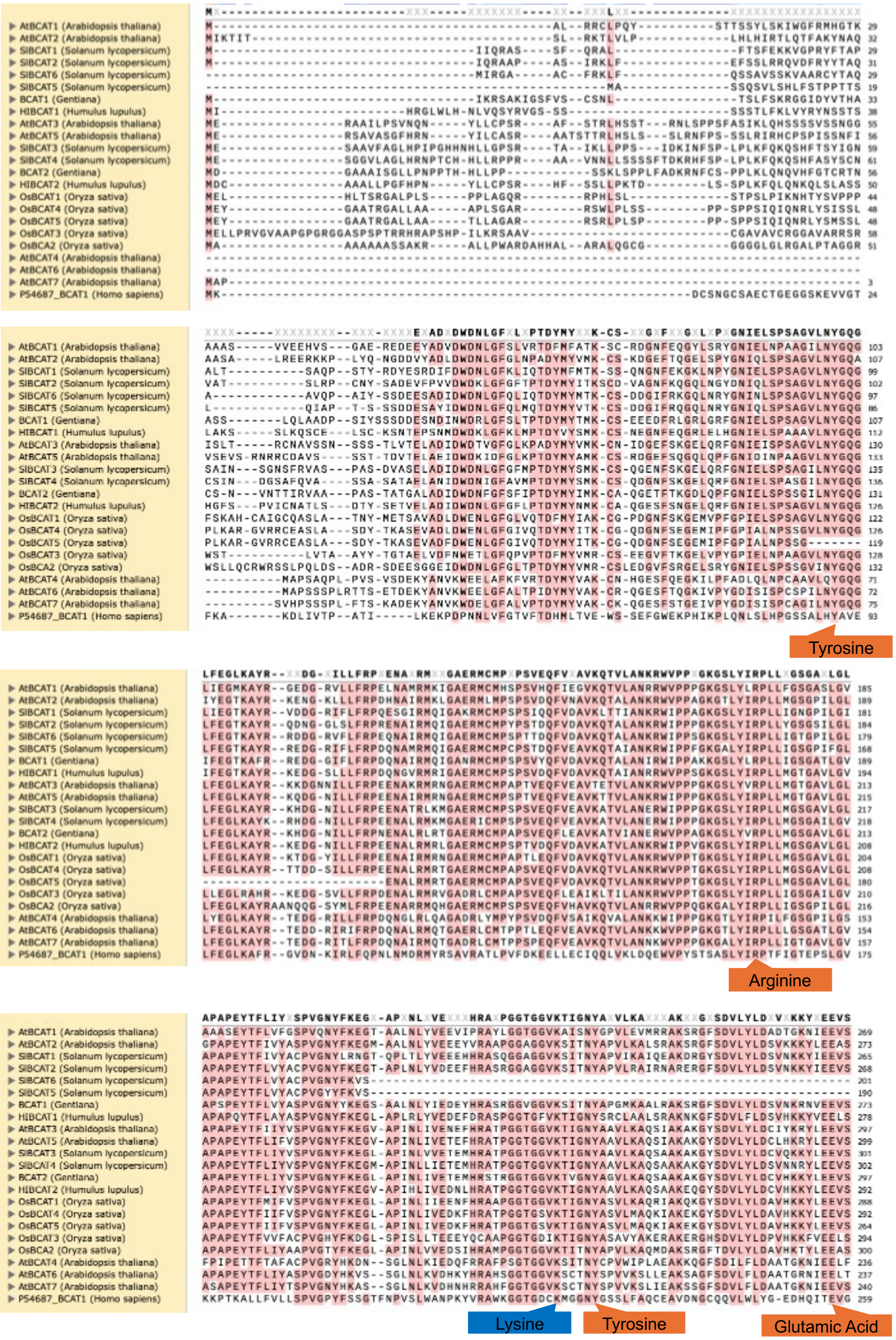
Tyrosine
Arginine
Lysine
Tyrosine
Glutamic Acid

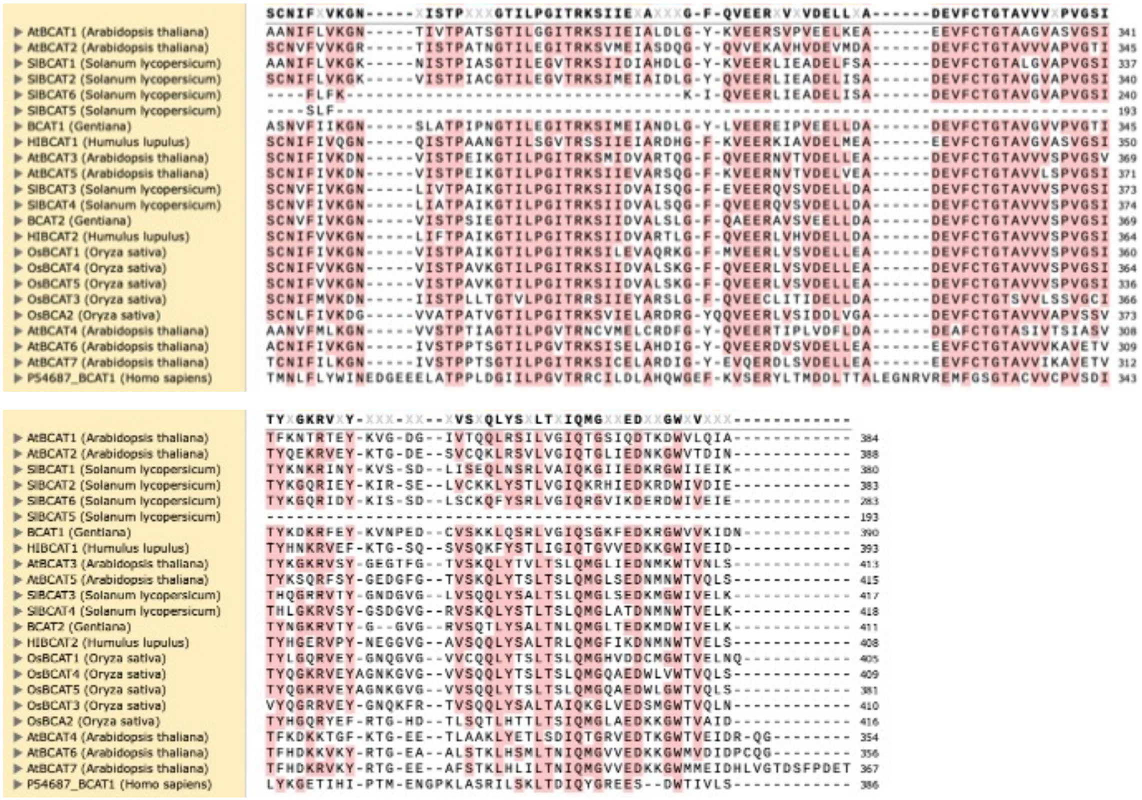

Supplementary Fig. S6 Alignment of BCAT amino acid sequences.

Conserved amino acid residues in plant BCATs are highlighted in red. Amino acids in the blue box indicate the pyridoxal-phosphate linking site; orange boxes highlight crucial amino acids identified through crystallization studies of the respective enzymes from *E. coli* and humans

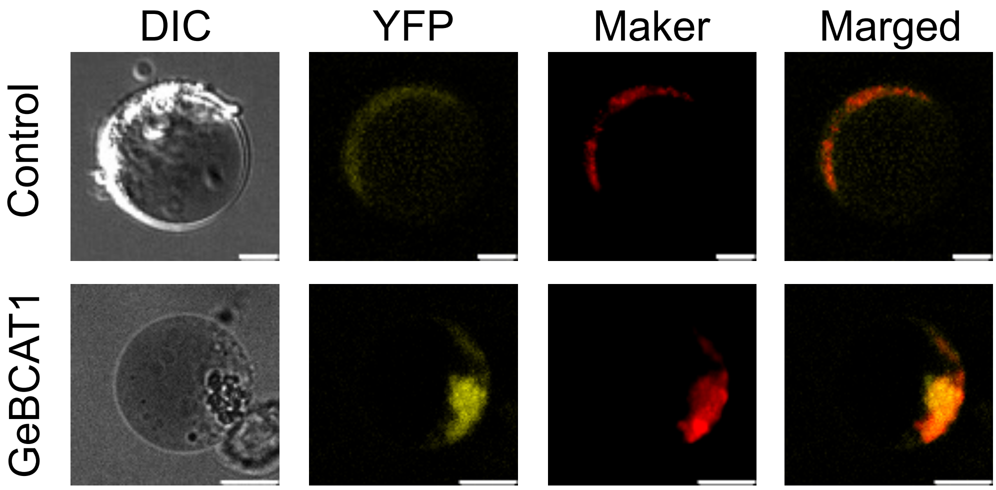

Supplementary Fig. S7 Subcellular localization of GeBCAT1 protein.
GeBCAT1 cDNA was fused to YFP at the C-terminus and transiently expressed in petal protoplasts of *G. triflora*. Yellow fluorescence indicates YFP-tagged GeBCAT1 ($\lambda_{ex}/\lambda_{em}$=514 nm/527 nm), and red fluorescence indicates mitochondrial staining using MitoRed ($\lambda_{ex}/\lambda_{em}$=560 nm/580 nm). Fluorescence signals were observed using fluorescence microscopy. DIC Differential interference contrast. Scale bars = 10 μm

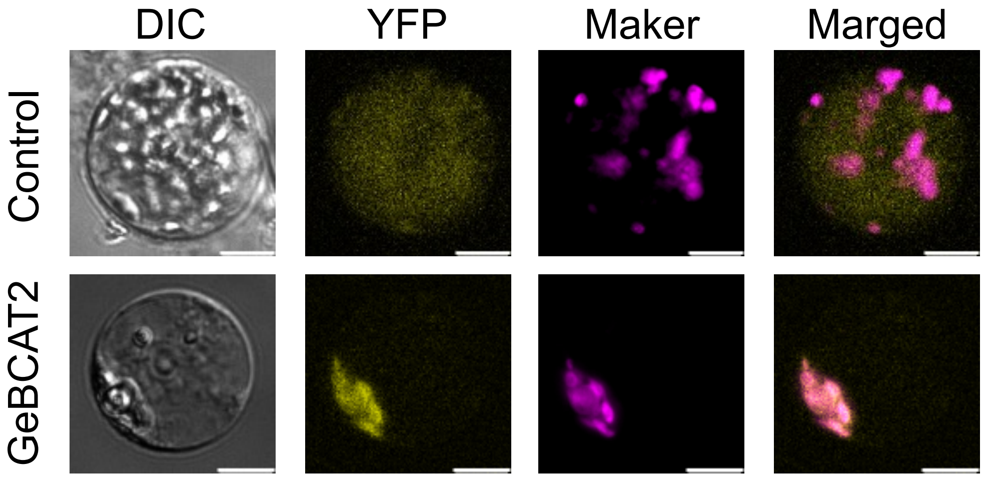

Supplementary Fig. S8 Subcellular localization of GeBCAT2 protein.
GeBCAT2 cDNA was fused to YFP at the C-terminus and transiently expressed in petal protoplasts of *G. triflora*. Yellow fluorescence indicates YFP-tagged GeBCAT2, and magenta fluorescence corresponds to chlorophyll autofluorescence ($\lambda_{ex}/\lambda_{em}$=543 nm/633 nm), marking the chloroplasts. Fluorescence signals were visualized using fluorescence microscopy. DIC Differential interference contrast. Scale bars = 10 μm

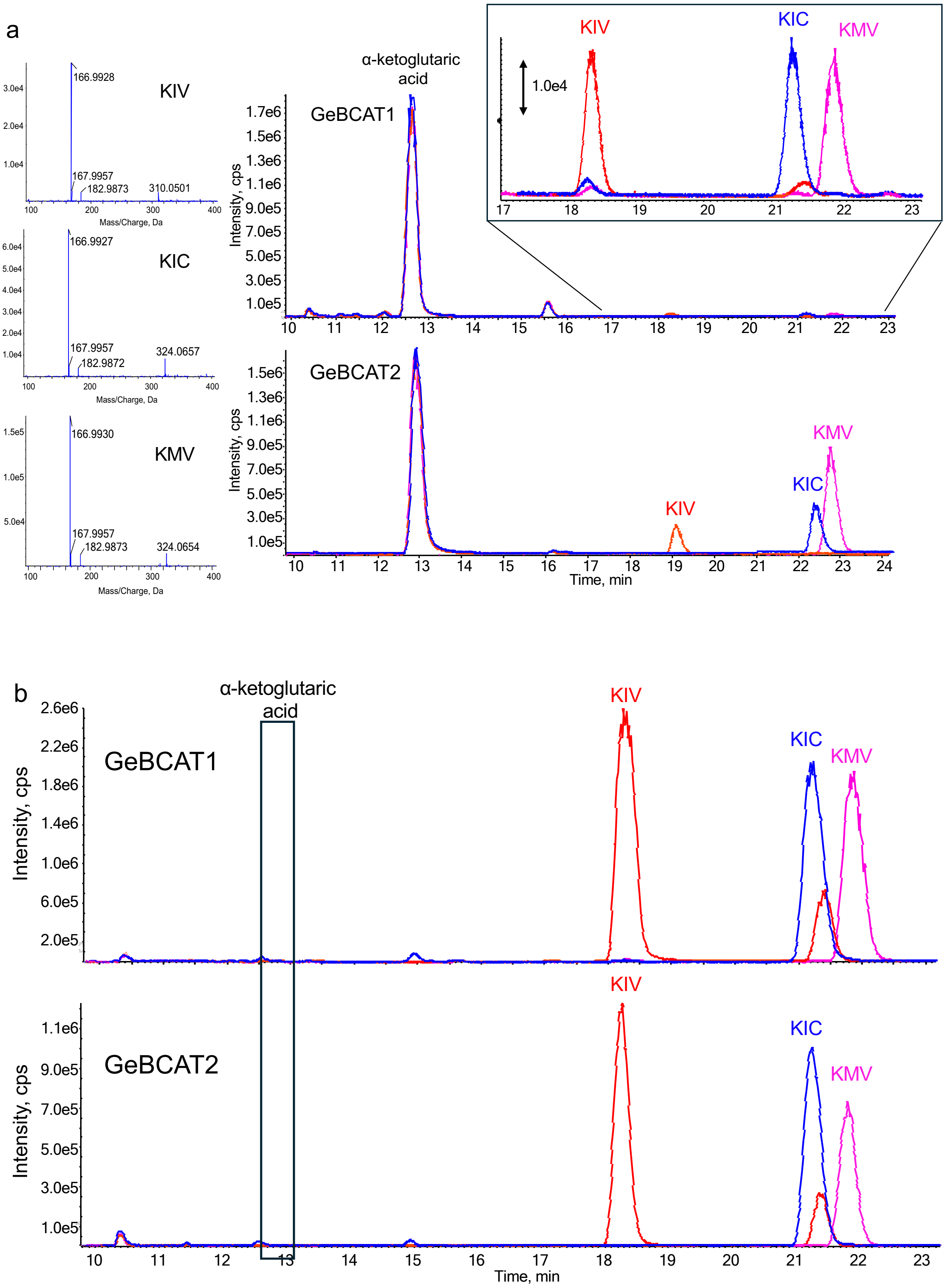

a
KIV
166.9928
167.9957
182.9873
310.0501
Mass/Charge, Da
KIC
166.9927
167.9957
182.9872
324.0657
KMV
166.9930
167.9957
182.9873
324.0654
α-ketoglutaric acid
GeBCAT1
GeBCAT2
Intensity, cps
Time, min
1.0e4
b

Supplementary Fig. S9 Functional analysis of branched-chain aminotransferase *in vitro.* **a** BCAAs catabolism and **b** BCAAs biosynthesis associated with GeBCAT1 and GeBCAT2. Chromatograms with the products formed by purified recombinant GeBCAT1 (upper) and recombinant GeBCAT2 (lower) from BCAAs obtained using LC-TOF-MS in negative enhanced product ion mode. The negative ion at *m/z* 167 corresponding to [C6F5]- was chosen as the parent ion in BCAAs biosynthesis and catabolism. In quantitative and qualitative analysis, the negative ions at *m/z* 324 corresponding to KIC and KMV [M-H+]- and *m/z* 310 corresponding to KIV [M-H+]- were chosen as the parent ions. KIC, KMV, and KIV were assigned by comparing the MS profiles and retention times of authentic KIC, KMV, and KIV. They were quantified using an externally constructed calibration curve

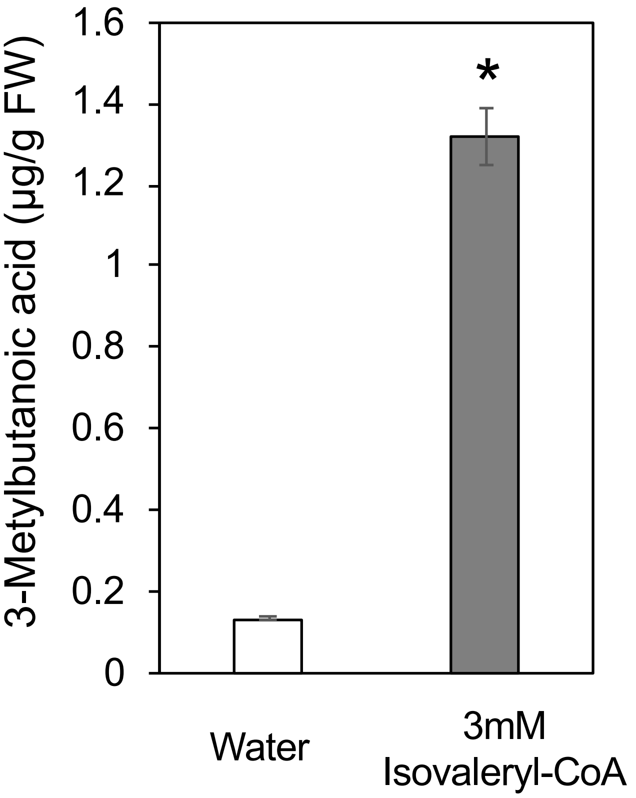

Supplementary Fig. S10 Volatile production following isovaleryl-CoA feeding in *G. triflora* petals.

Changes in branched-chain VOCs after feeding petals with isovaleryl-CoA and overnight incubation. Values are presented as percentages of volatile emissions relative to water-fed controls ± SDs (n = 3). Asterisks marking columns indicate statistically significant changes based on Student's t-test ($P < 0.05$)

Table S1. Primers used in this study.

Primer name	Primer sequence (5'–3')	Use
GtBCAT1-F	ATGATTAAGAGAAGTGCGAAAATTG	cds
GtBCAT1-R	CTAATTGTCGATTTTGACAACCCAA	cds
GtBCAT2-F	ATGGACGGCGCCGCCGCCATCTC	cds
GtBCAT2-R	TTATTTTAGCTCAACAATCCAATCC	cds
r-GtBCAT1-F	ACAAGTTTGTACAAAAAAGCAGGCTTCATGTACGTAACACAT	Expression of recombinant protein
r-GtBCAT1-R	ACCACTTTGTACAAGAAAGCTGGGTCATTGTCGATTTTGAC	Expression of recombinant protein
r-GtBCAT2-F	ACAAGTTTGTACAAAAAAGCAGGCTTCATGACTAATTGCAGT	Expression of recombinant protein
r-GtBCAT2-R	ACCACTTTGTACAAGAAAGCTGGGTCTTTTAGCTCAACAAT	Expression of recombinant protein
q-GtBCAT1-F	GTGTATGCTTCGCCGGTTGG	qRT-PCR
q-GtBCAT1-R	GCTTTTAGTGCTGCTTTCATG	qRT-PCR
q-GtBCAT2-F	CTGTAATAGCAAACGAAAGATG	qRT-PCR
q-GtBCAT2-R	GGAAATAATTACCGACAGGTG	qRT-PCR
UBQ2-F	TGGTGGTTTCTGAATGTTGTCTTCT	qRT-PCR
UBQ2-R	CACATCCTGGCATCACAAACTC	qRT-PCR
